# Supplementary material for: Chromothripsis during telomere crisis is independent of NHEJ, and consistent with a replicative origin
Source: Genome Res. 2019 May;29(5):737–49. doi: 10.1101/gr.240705.118 (PMC6499312; doi:10.1101/gr.240705.118)
Supplement: Supplemental Material [file supp_gr.240705.118_Supplemental_file_1.zip › contigs/annotated_contigs/DB89/contig.2.DB89_length_499_mean_cov_7.51503006012.docx]

**DB89_length_499_mean_cov_7.51503006012**

```
TGAATAAACAGTTTCAAGTGATAAATCAGGAATCTAGGTCACATATGTATTAGACATGTATTCTTGTGTATATGGACATTCTAATAACC
 >chrX:8963932-8964099 + E=7e-80 p=1e-02
CATATCAATGTGGCTTTTAACAATTCTTTCCTTTTTAAAAAATTGGATTAATATTTACTCTATAAACATTTATTGAG|A|TCAGAAGGT
                                                                              >chr10:83
AGGAGAAGTCATATTATAAGTTAATATTGTTTCAATCCAAAATCTCTTTAATTTTAACATTTCTATGTTAATTTGATTTATGGGTGTTG
930615-83930948 + E=6e-189
TCAGATTGTTTTATGCAGTCTGAGATCATTTGTCTTCATGTGAAGGAGCTGAAGACATTAATATTGATTATGATAATAGATACTCTGGC

TTTATTAGTGTCATTATTTTCTATTTTCTTTCATTTTCTTTTCCTCCCATTTTTGTTTAATTCTCATTTAGTCTCCTGGTGCAATTCAA

TCAATGTATTTGGTATGAAGTATTTCAAAAATAGAAGTCCCATTTTTGCATAAATT
```
